# Optimized Method for Establishing Primary Human Mesothelial Cell Cultures Preserving Epithelial Phenotype

**DOI:** 10.3390/biom15121669

**Published:** 2025-11-30

**Authors:** Evdokiya Kuznetsova, Nadezhda Bakalenko, Liana Gaifullina, Mikhail Atyukov, Konstantin Dergilev, Irina Beloglazova, Anna Malashicheva

**Affiliations:** 1 Institute of Cytology, Russian Academy of Sciences, 194064 St-Petersburg, Russiabakalenko@gmail.com (N.B.); lianagaifullina.ya@gmail.com (L.G.); 2 St. Petersburg State Budgetary Institution of Health Care “City Multidisciplinary Hospital No. 2”, 194354 St-Petersburg, Russia; 3 Institute of Experimental Cardiology Named After Academician V.N. Smirnov, Federal State Budgetary Institution National Medical Research Center of Cardiology Named After Academician E.I. Chazov, Ministry of Health of the Russian Federation, 121552 Moscow, Russia; kvdergilev@cardio.ru (K.D.);

**Keywords:** mesothelial cells (MCs), primary cell cultures, mesothelial-to-mesenchymal transition (MMT), in vitro cell models

## Abstract

Mesothelial cells (MCs) are highly relevant for studying the pathogenesis of serosal diseases, fibrosis, inflammation, and tumor progression. However, the isolation and maintenance of an epithelial-like phenotype of MCs in vitro remain methodologically challenging due to their tendency to undergo mesothelial-to-mesenchymal transition (MMT). In this work, we propose a combined protocol utilizing collagen IV coating, conditioned medium, and short-term ROCK inhibitor treatment, which improves cell survival. This approach enables the establishment of primary human cultures suitable for investigating mesothelial cell functional activity and for assessing the efficacy of potential therapeutic strategies.

## 1. Introduction

The mesothelium lines the serosal cavities of the body and covers internal organs, including the pericardium, pleural cavity, peritoneum, and mesentery. For a long time, mesothelial cells (MCs) were regarded as a passive barrier that provides mechanical protection and reduces friction between organs through the secretion of mucus and lubricating substances [1]. However, evidence accumulated over the past decades has firmly established their role as active regulators of various physiological and pathological processes. MCs control transport processes between serosal cavities and internal organs, including the movement of fluids, ions, and possibly cellular components [1]. Their immunoinflammatory function involves pathogen recognition, secretion of proinflammatory cytokines and chemokines, antigen presentation, and activation of submesothelial and immune cells [2,3,4]. In addition, they contribute to extracellular matrix (ECM) homeostasis by synthesizing its components, regulating their degradation through matrix metalloproteinases and their inhibitors, and maintaining the balance between fibrin deposition and degradation [4]. Mesothelial cells exhibit high phenotypic plasticity and the ability to undergo mesothelial-to-mesenchymal transition (MMT), a process accompanied by the loss of epithelial features, upregulation of mesenchymal markers, and increased secretory activity.

MCs play a pivotal role in inflammation, fibrosis, tumor progression, and tissue regeneration by shaping the local microenvironment and secreting a wide range of bioactive mediators [1]. Their involvement in these processes makes MCs a valuable model for studying the pathogenesis of serosal diseases and intercellular communication. Moreover, inhibition of signaling pathways that induce mesothelial-to-mesenchymal transition (MMT) is considered a promising therapeutic strategy [5]. In this context, primary human mesothelial cell cultures serve as an indispensable model for investigating molecular mechanisms and evaluating potential therapeutic approaches [6]. Growing interest in reliable mesothelial biomarkers has also emerged in recent years, driven by advances in omics technologies. This highlights the need for high-quality primary mesothelial cultures that allow not only verification of their in vitro phenotype but also the investigation of marker variability under pathological conditions, environmental influences, and therapeutic interventions [7].

Although mesothelial cell lines exist and are widely used, studies employing primary cultures offer distinct advantages. Primary mesothelial cells retain their natural phenotype, including morphology and marker expression, making them more representative of in vivo conditions. They also preserve donor-specific variability, allowing the detection of interindividual differences that are lost in immortalized cell lines [8]. Moreover, primary cultures provide a more physiologically relevant response to experimental stimuli and pharmacological agents [9]. In contrast, continuous cell lines are prone to genetic drift, mutations, and phenotypic alterations during long-term cultivation, which may affect their signaling networks and cellular behavior [10].

Establishing and maintaining an epithelial-like phenotype of MCs in vitro remain methodologically challenging tasks, as there is still no standardized and reproducible protocol for their isolation and culture. Mesothelial cells can be obtained either from exfoliated cells present in serous fluids or through mechanical and enzymatic tissue dissociation methods [11,12]. In the case of pleural MCs, postoperative pleural tissue fragments obtained from patients undergoing pleurectomy for primary spontaneous pneumothorax without associated pathology represent an accessible and reliable source. Several approaches for isolating pleural mesothelial cells have been described in the literature. One common method involves enzymatic digestion of tissue explants using 0.25% trypsin-EDTA following mechanical dissociation, with subsequent culture on uncoated plastic and without inhibitors of pathways associated with mesothelial-to-mesenchymal transition (MMT) [11,13]. Under these conditions, a gradual loss of epithelial-like morphology is typically observed by the third passage, accompanied by a decline in specific mesothelial marker expression and fibroblast contamination [13,14,15,16]. Alternative protocols employing collagenase digestion, alone or in combination with trypsin, have also been tested, although such approaches tend to increase fibroblast contamination [17]. Optimization efforts have mainly focused on reducing enzymatic exposure time and employing milder digestion procedures [18]. It is important to note that, due to these limitations, early-passage cells (no later than passage 3) are generally recommended for experimental use.

Under in vitro culture conditions, MCs are prone to MMT and rapid differentiation toward a fibroblast-like phenotype due to stress factors associated with isolation and passaging. They also exhibit reduced survival [19]. Several studies have proposed approaches to improve the efficiency of mesothelial cell isolation and to maintain an epithelial-like phenotype; however, no protocol has yet combined all these improvements.

In this study, we propose an optimized protocol for MCs isolation and culture that combines collagen IV coating, the ROCK inhibitor Y-27632, and conditioned medium. This approach enables primary mesothelial cells to maintain a stable epithelial-like phenotype in vitro.

## 2. Materials and Methods

### 2.1. Primary MCs Isolation and Culture

Mesothelial cell cultures were derived from the pleura of patients with primary spontaneous pneumothorax. Tissue samples were washed with PBS containing 2% antibiotics, mechanically dissociated into fragments measuring ~2–3 mm using a sterile scalpel, and incubated in 0.05% trypsin-EDTA (Gibco, St. Louis, MO, USA) at 37 °C for 15–20 min. The tissue fragments were then subjected to gentle mechanical dissociation using a 5 mL serological pipette. The resulting cell suspension was filtered through 70 µm cell strainers (Corning, St. Louis, MO, USA) to eliminate tissue fragments and obtain a homogeneous population. Cells were pelleted by centrifugation (300× *g*, 5 min) and seeded into 25 cm^2^ flasks coated with collagen IV (10 µg/cm^2^) in RPMI medium (Gibco) supplemented with 5% fetal bovine serum (FBS, Cytiva) St. Louis, MO, USA, 100 U/mL penicillin, 100 µg/mL streptomycin, and 2 mmol/L L-glutamine (Gibco, St. Louis, MO, USA). The following day, cultures were washed with PBS, and the medium was replaced to remove dead cells and residual blood. Cells were maintained at 37 °C in 5% CO_2_, with partial medium changes 2–3 times a week. Passaging was performed at 90–95% confluence using TrypLE (Gibco, St. Louis, MO, USA) in the presence of ROCK inhibitor (Y-27632, 5 µM, Tocris, Bristol, United Kingdom), which was gradually withdrawn, and supplemented with conditioned medium (CM) from the mesothelial cultures. The CM was collected from primary mesothelial cells obtained from the same donor as the recipient culture. Donor cultures were grown from passage 0 to 70–80% confluence and incubated with fresh medium for 48–72 h. CM was collected immediately prior to passage and centrifuged at 300× *g* for 5 min to remove cell debris. CM aliquots were stored at –80 °C for long-term use (up to 6–12 months) or at –20 °C for short-term use (up to 1–2 months). For the gradual removal of the ROCK inhibitor and CM after cell seeding, the medium was replaced in stages in a single passage: 24 h after seeding, half of the medium was replaced with fresh medium; 48 h after seeding, two-thirds of the medium was replaced; 72 h after seeding, the medium was completely replaced. At this stage, the cells typically reached 70–80% confluence. This schedule represents a gradual removal of both the ROCK inhibitor and the conditioned medium while maintaining cell viability and the mesothelial phenotype. Cultures retained stable proliferative capacity up to passages 5–6. All experiments were performed using cells derived from three independent donors (n = 3). Data are presented as mean ± standard deviation (SD).

### 2.2. RNA Isolation and Real-Time PCR (RT-PCR)

Total RNA was extracted using TRIzol reagent (Evrogen, Moscow, Russia) according to the manufacturer’s instructions. cDNA synthesis was performed using MMLV reverse transcriptase and random decamer primers (Evrogen, Russia). Quantitative gene expression analysis was carried out by real-time PCR using SYBR Green PCR Mastermix (Evrogen, Russia) on a LightCycler 96 system (Roche, Basel, Switzerland). Amplification was performed with gene-specific forward and reverse primers designed using Oligo 6 software (Molecular Biology Insights, USA). mRNA levels were normalized to glyceraldehyde-3-phosphate dehydrogenase (GAPDH), and relative expression changes were calculated using the 2^−ΔΔCt^ method. Primers used for RT-PCR were as follows (Table 1).

Statistical analyses were performed using STATISTICA 10.0 and GraphPad Prism 9.0. For comparisons between two independent groups, Student’s *t*-test was applied.

### 2.3. Immunocytochemistry

Cells for immunophenotyping were cultured on coverslips and fixed with 4% paraformaldehyde. The detailed staining protocol has been described previously [20]. Primary antibodies used were anti-KRT5 (ab75869), pan-cytokeratin (ab9377), mesothelin (MSLN, MA5-42914), and podoplanin (PDPN, ab236529). Fluorescent secondary antibodies (Alexa Fluor 488/594) were applied, and nuclei were counterstained with DAPI (Ibidi GmbH, Gewerbehof Gräfelfing, Germany). Imaging was performed on an Olympus FV3000 confocal microscope (Olimpus, Suva, Japan) at 40× magnification using the integrated software.

## 3. Results

**Figure 1:**
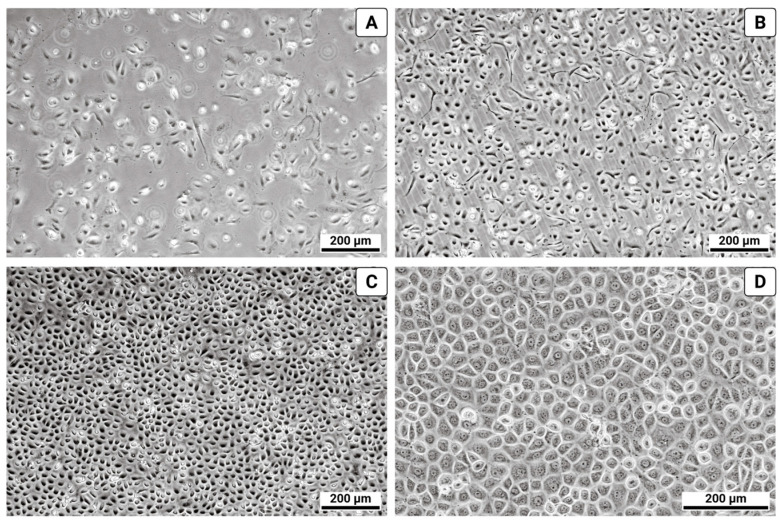
Morphology of primary human pleural mesothelial cells (light microscopy). (**A**) 48 h after tissue isolation; (**B**) 72 h after isolation; (**C**) 120 h after isolation; (**D**) 120 h after isolation at higher magnification.

During the culture of cells isolated from the pleura, a homogeneous monolayer formed within 3–5 days, displaying characteristic polygonal morphology (“cobblestone” pattern) indicative of the epithelial-like mesothelial phenotype (Figure 1).

**Figure 2:**
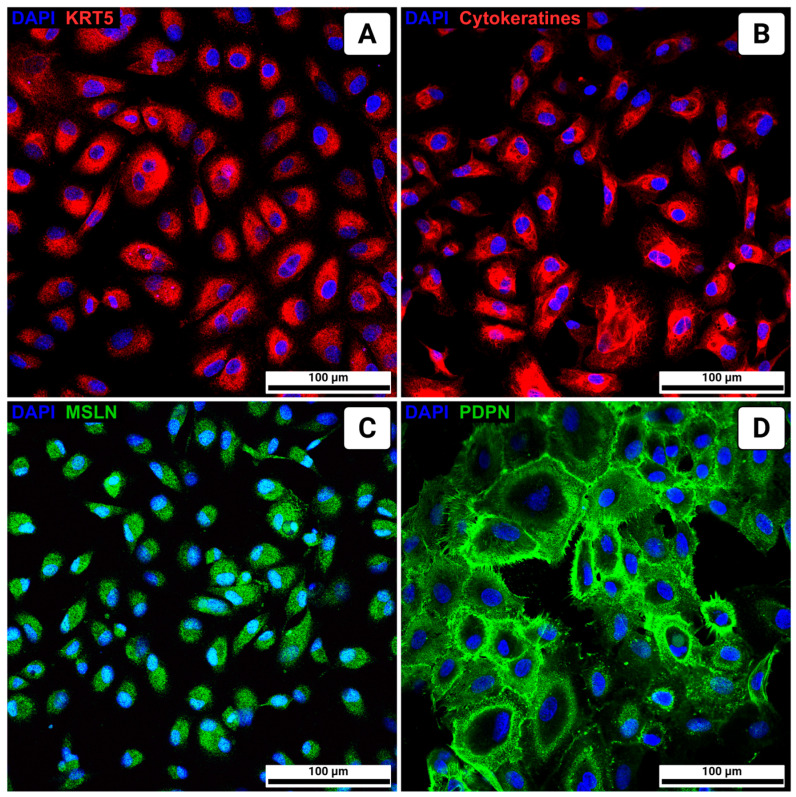
Immunocytochemical staining of key mesothelial cell markers. Nuclei are stained blue (DAPI). (**A**) keratin 5 (KRT5) in red, (**B**) pan-cytokeratin (cytokeratins) in red, (**C**) mesothelin (MSLN) in green, (**D**) podoplanin (PDPN) in green.

**Figure 3:**
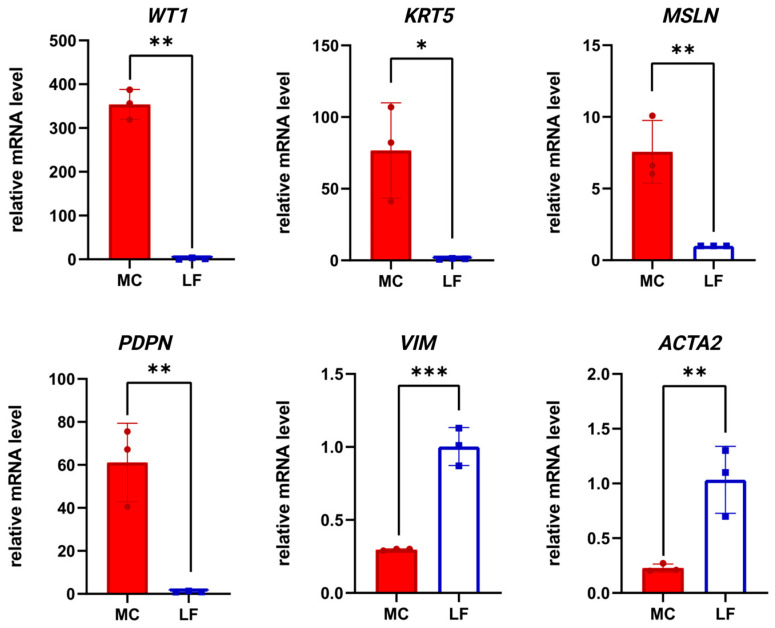
Gene expression analysis by RT-PCR. X-axis: analyzed genes (*WT1*, *KRT5*, *MSLN*, *PDPN*, *VIM*, *ACTA2*); Y-axis: relative gene expression normalized to GAPDH. Left bars: mesothelial cells (MC); right bars: lung fibroblasts (LF); Data are shown as mean ± SD; * *p* ≤ 0.05; ** *p* ≤ 0.01; *** *p* ≤ 0.001, Student’s *t*-test.

**Figure 4:**
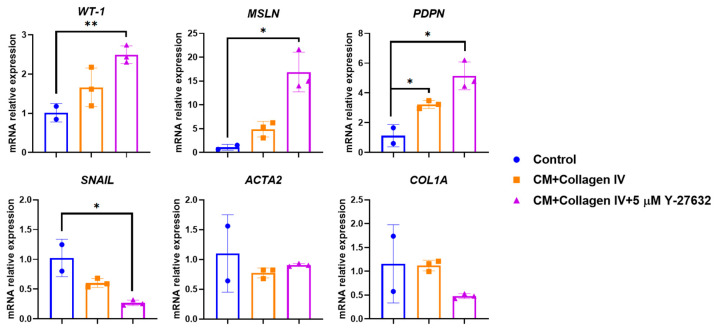
Gene expression analysis by RT-PCR. X-axis: analyzed genes (*WT1*, *MSLN*, *PDPN*, *SNAIL, ACTA2, COL1A*); Y-axis: relative gene expression normalized to GAPDH. Conditioned medium (CM); Data are shown as mean ± SD; * *p* ≤ 0.05; ** *p* ≤ 0.01, Student’s *t*-test.

Immunocytochemical analysis revealed expression of keratin 5 (KRT5), pan-cytokeratin, mesothelin (MSLN), and podoplanin (PDPN) (Figure 2). RT-PCR confirmed the expression of *WT1*, *KRT5*, *MSLN*, and *PDPN*, whereas the levels of mesenchymal markers (*VIM*, *ACTA2*) were significantly lower compared to lung fibroblasts. These results confirm the mesothelial phenotype of the cultured cells (Figure 3). To confirm the reliability of the optimized protocol, we compared gene expression profiles between mesothelial cells on passage 3 cultured on collagen IV-coated plastic with the addition of ROCK inhibitor and conditioned medium, and cells grown under non-optimized conditions. As shown in Figure 4, cells cultured in the optimized system maintained higher expression of mesothelial markers (*WT1*, *MSLN*, *PDPN*) and showed consistently low levels of SNAIL gene expression (a marker of MMT). These results demonstrate that the combination of collagen IV coating, ROCK inhibition, and conditioned medium is more effective at maintaining the mesothelial phenotype over time.

**Figure 5:**
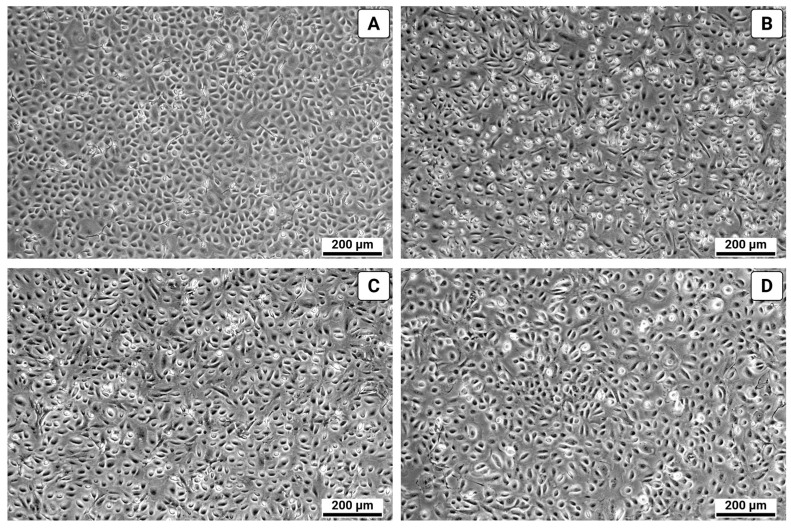
Morphology of mesothelial cells after passaging (passage 2) under different conditions. (**A**) 24 h after passaging in the presence of ROCK inhibitor (5 µM); (**B**) 48 h after passaging (24 h after medium replacement); (**C**) 72 h after passaging (48 h after medium replacement); (**D**) 72 h after passaging with gradual medium replacement.

**Figure 6:**
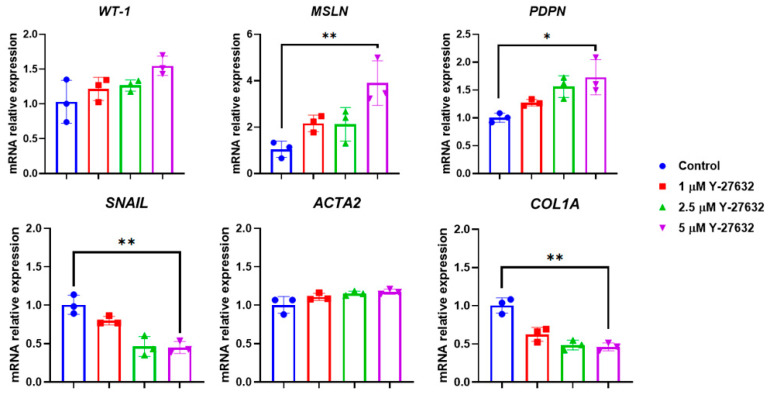
Gene expression analysis (RT-PCR) of mesothelial cells after passaging (passage 2) under different conditions. X-axis: analyzed genes (*WT1*, *MSLN*, *PDPN*, *SNAIL, ACTA2, COL1A*); Y-axis: relative gene expression normalized to GAPDH. Data are shown as mean ± SD; * *p* ≤ 0.05; ** *p* ≤ 0.01, Student’s *t*-test.

After passaging in the presence of ROCK inhibitor, cells retained their typical mesothelial morphology (Figure 5A). However, abrupt medium replacement after passaging and removal of the ROCK inhibitor, along with conditioned medium, led to a more fibroblast-like phenotype within 24 h (Figure 5B), which became more pronounced by 72 h (Figure 5C), indicative of MMT. Nevertheless, the use of ROCK inhibitor significantly improved cell survival. Therefore, a stepwise withdrawal of the inhibitor with gradual medium replacement (first 12, then 23, and finally completely as described) was adopted to preserve the epithelial-like phenotype. This approach markedly maintained the mesothelial phenotype and facilitated cell adaptation during passaging (Figure 5D). In addition to morphological observations, we performed RT-PCR analysis. Cells maintained under optimized conditions at a ROCK inhibitor concentration of 5 μM showed higher expression of mesothelial markers (WT1, MSLN, PDPN) and lower levels of MMT/mesenchymal genes (SNAIL, ACTA2, COL1A). In contrast, non-optimized conditions were associated with decreased expression of mesothelial markers and increased levels of mesenchymal genes (Figure 6).

## 4. Discussion

Mesothelial cells (MCs) play a key role in maintaining the homeostasis of serous membranes and are involved in regeneration, fibrosis, and tumor pathogenesis. Studying them in vitro requires primary human cultures that preserve the morphological and molecular characteristics of the mesothelium. Their high sensitivity to microenvironmental changes and inherent subpopulation heterogeneity impose specific requirements on isolation and culture methods. Unlike tissues affected by chronic lung disease, PSP is not associated with persistent inflammation or fibrosis, which minimizes extraneous factors and provides a reliable source of “normal-like” mesothelial cells. Although the protocol has not yet been applied to fibrotic or malignant pleural tissues, extending these optimized conditions to samples with more pronounced pathological changes may help determine its broader applicability.

To enhance the efficiency of cell isolation from tissue, a combination of mechanical and enzymatic dissociation with low enzyme concentrations is recommended [21]. This is due both to the high responsiveness of MCs to microenvironmental changes and their initial subpopulation heterogeneity [1,14].

In this study, a combined approach was applied, including coating culture vessels with collagen IV, supplementing with conditioned medium from mesothelial cells, and transient use of ROCK inhibitor with gradual dose reduction (“weaning”). This comprehensive strategy aims to preserve phenotype and improve culture stability. To maintain the mesothelial phenotype in vitro, coatings mimicking the basement membrane, such as collagen IV, laminin, and Matrigel, are commonly used [22]. In particular, collagen IV enhances cell adhesion to the culture surface [23]. Conditioned medium provides factors that support epithelial morphology and stimulate cell proliferation, including low levels of EGF family ligands, HGF, IGF-binding proteins, and paracrine mediators that help maintain junction integrity (e.g., IL-1 receptor antagonists, CXCL chemokines) [24,25,26]. Proteomic data further indicate the presence of ECM-associated proteins such as fibronectin, collagens, laminin, thrombospondin-1, and perlecan, along with moderate levels of IL-8, GRO-α/β, MIF, and complement C3. Collectively, these growth factors, survival signals, and ECM-associated signals likely help protect cells during early adaptation to culture, thereby reducing their susceptibility to MMT [27]. Early-stage use of ROCK inhibitor (Y-27632) increases cell survival and prevents stress-induced alterations [28,29,30].

It is known that epithelial-to-mesenchymal transition (EMT) may only be partially reversible upon inhibition of the TGF-β signaling pathway and/or ROCK suppression, as prolonged stimulation and transformation can trigger irreversible epigenetic changes [31]. Prolonged ROCK inhibitor exposure may reduce migratory and reparative activity, alter signaling cascades, and suppress proliferation [29,32]. Therefore, a transient application with gradual dose reduction is considered optimal [32]. In addition to ROCK inhibition, other TGF-β pathway blockers, such as SB431542, can be used to stabilize the phenotype [14]. However, the effect of their combined use in vitro remains insufficiently studied, making minimal intervention the preferred strategy to preserve the physiological response of primary cell cultures.

Thus, the application of this multi-step strategy improved the preservation of the normal mesothelial phenotype and its proliferative capacity in vitro. The primary cultures of pleural mesothelial cells generated in this study express the key markers characteristic of this cell type. Immunocytochemical analysis revealed expression of KRT5, pan-cytokeratin, MSLN, and PDPN. Real-time PCR confirmed elevated expression of WT1 [33], KRT5 [34], MSLN [35], and PDPN [36] compared to lung fibroblasts. These findings demonstrate that the cultures retain the phenotype of normal mesothelium and represent a reliable model for further in vitro experimental studies. Although morphology and RT-PCR confirm a decrease in mesenchymal marker expression under optimized conditions, confirmation by immunostaining with vimentin or α-SMA would further strengthen these results. This issue will be addressed in future work.

Nevertheless, several questions remain to be addressed. The tissue origin of mesothelial cells is critical: the mesothelium of the peritoneum, pleura, and pericardium exhibits organ-specific differences in transcriptional profiles and responsiveness, which affect cellular behavior in culture. Moreover, although mesothelial cells were previously considered a homogeneous epithelial layer, recent data—including single-cell transcriptomics—reveal pronounced cellular heterogeneity [14]. Currently, there is no complete map of subpopulations or knowledge of which subpopulations truly contribute to fibrosis, regeneration, or tumor support. If subpopulations perform distinct functions, therapeutic interventions will need to be precisely targeted. Advancing this field will require determining which tissue-resident subpopulations are maintained in culture, which are lost, and whether specific markers exist for selective isolation of these subpopulations. Future studies could use flow cytometry with markers such as MSLN, PDPN, and CD44 to characterize the distribution of mesothelial cell subpopulations in different passages, which would provide a deeper understanding of the heterogeneity and stability of the cultures obtained.

## 5. Conclusions

In conclusion, the optimized protocol combining collagen IV coating, conditioned medium, and transient ROCK inhibitor (Y-27632) treatment enables efficient isolation and long-term maintenance of primary human pleural mesothelial cells with a stable epithelial-like phenotype. This approach improves cell survival, reduces stress-induced mesothelial-to-mesenchymal transition, and minimizes fibroblast contamination. The cultures represent a reliable and physiologically relevant model for investigating mesothelial biology, molecular mechanisms of serosal diseases, and testing potential therapeutic strategies aimed at modulating mesothelial plasticity and fibrosis.

## Figures and Tables

**Table 1:** Primers for RT-PCR.

Genes	Primers
glyceraldehyde-3-phosphate dehydrogenase (GAPDH)	F: 5′-ACAATTTGCTATCGTGGAAGG-3′ R: 5′-CCTTCCACGATAGCAAATTGT-3′
mesothelin (MSLN)	F: 5′-CCTGAGGACATTCGCAAGTGGA-3′ R: 5′-CTTGAGGACATTCGCAAGTGGA-3′
keratin 5 (KRT5)	F: 5′-CAGTGGAGAAGGAGTTGGACC-3′ R: 5′-TGCTGCTGGAGTAGTAGCTT-3′
actin alpha 2 (ACTA2) or alpha smooth muscle actin (α-SMA)	F: 5′-CTATGCCTCTGGACGCACAACTCAGATC-3′ R: 5′-CAGACGCATATGATGGCA-3′
Wilms tumor protein 1 (WT-1)	F: 5′-CGAGAGCGATAACCACACAACG-3′ R: 5′-GTCTCAGATGCCGACCGTACAA-3′
vimentin (VIM)	F: 5′-GAGAACTTTGCCGTTGAAGC-3′ R: 5′-GCTTCCTGTAGGTGGCAATC-3′

## Data Availability

All data related to this work can be made available upon request to the corresponding authors.
